# Two Crystal Forms of a Hydrated 2:1 β-Cyclodextrin Fluconazole Complex: Single Crystal X-ray Structures, Dehydration Profiles, and Conditions for Their Individual Isolation

**DOI:** 10.3390/molecules26154427

**Published:** 2021-07-22

**Authors:** Andrea Sala, Zakiena Hoossen, Alessia Bacchi, Mino R. Caira

**Affiliations:** 1 Centre for Supramolecular Chemistry Research, Department of Chemistry, University of Cape Town, Rondebosch 7701, South Africa; andrea.sala@unipr.it (A.S.); hsszak005@myuct.ac.za (Z.H.); 2 Department of Chemical, Life and Environmental Sustainability Sciences, Università Degli Studi di Parma, Viale delle Scienze, 17A, 43124 Parma, Italy; alessia.bacchi@unipr.it

**Keywords:** pharmaceutical solids, cyclodextrins, fluconazole, inclusion complexes, dehydration, thermal analysis, X-ray diffraction, crystal structure, stability, phase solubility

## Abstract

Inclusion complexes between cyclodextrins (CDs) and active pharmaceutical ingredients (APIs) have potential for pharmaceutical formulation. Since crystallization of a given complex may result in the isolation of multiple crystal forms, it is essential to characterize these forms with respect to their structures and physicochemical properties to optimize pharmaceutical candidate selection. Here, we report the preparation and characterization of two crystallographically distinct hydrated forms of an inclusion complex between β-cyclodextrin (β-CD) and the antifungal API fluconazole (FLU) as well as temperature–concentration conditions required for their individual isolation. Determination of crystal water contents was achieved using thermoanalytical methods. X-ray analyses revealed distinct structural differences between the triclinic (TBCDFLU, space group P1) and monoclinic (MBCDFLU, space group C2) crystal forms. Removal of the crystals from their mother liquors led to rapid dehydration of the MBCDFLU crystal, while the TBCDFLU crystal was stable, a result that could be reconciled with the distinct packing arrangements in the respective crystals. This study highlights (a) the importance of identifying possible multiple forms of a cyclodextrin API complex and controlling the crystallization conditions, and (b) the need to characterize such crystal forms to determine the extent to which their physicochemical properties may differ.

## 1. Introduction

Extending the solid-state landscape of active pharmaceutical ingredients (APIs) and promising drug candidates via the generation of new forms (e.g., polymorphs and multicomponent species such as solvates, inclusion complexes, salts, co-crystals, and eutectics) continues to be a vibrant pursuit in both academia and in the pharmaceutical industry [1,2,3]. Notably, these types of ‘supramolecular derivatives’ contain the API intact, with retention of its bioactivity since no covalent bonds are created or broken during their synthesis. There are numerous critical and pharmaceutically relevant properties of a given API or drug candidate that can be modified via this approach to their beneficiation. Well-known potential advantages that may ensue from the availability of a new solid form of an API include improved solubility (for enhanced absorption), greater chemical stability (preventing API degradation), reduced hygroscopicity, higher compressibility (affecting tabletability), and an increase in thermal stability (via melting point modulation) [4]. Moreover, new solid forms of APIs of the types listed above can be rationally designed using crystal engineering principles and they are often prepared by relatively simple, economical, and efficient procedures such as recrystallization, co-precipitation, and co-grinding or kneading of the API with one or more biocompatible compounds [5,6,7]. Exploiting the multiplicity of solid forms that could be generated for a given API can thus lead to innovative new materials with potential for novel formulations. Such materials could effect significant improvements in drug delivery and a reduction in manufacturing costs.

**Figure 1:**
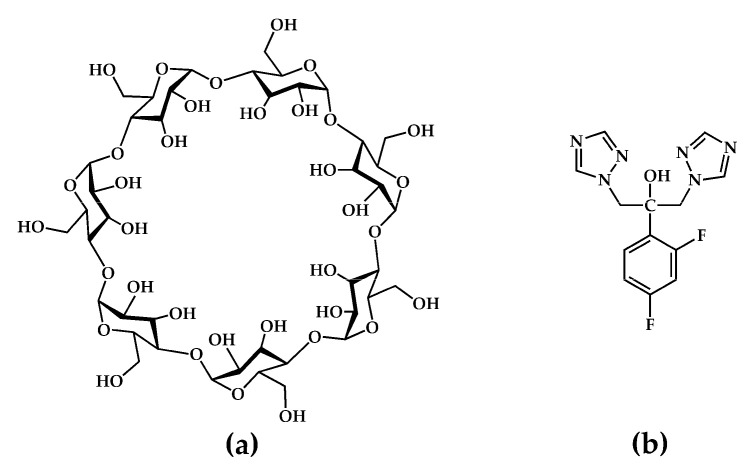
Chemical structures of (**a**) the host compound β-cyclodextrin and (**b**) the API fluconazole.

In principle, each new solid form of a given API displays unique physicochemical properties. Thus, an important aspect of the preparation and characterization of any new solid species is the need to establish whether a given set of starting materials gives rise to more than one crystal form, and if so, to isolate and characterize such multiple products and determine their specific preparative conditions for ensuring reliable procedures for their future isolation. This report highlights such an outcome by focusing on the synthesis and physicochemical characterization of two distinct crystalline inclusion complexes formed between the host compound β-cyclodextrin (β-CD) and the triazole antifungal API fluconazole (2-(2,4-difluorophenyl)-1,3-bis(1,2,4-triazol-1-yl)propan-2-ol, FLU hereinafter) (Figure 1).

β-CD, a prominent member of the native (natural/underivatized) cyclodextrins, is a cyclic oligosaccharide composed of seven α-1,4-linked D-glucopyranose units, frequently employed as a complexing agent to solubilize hydrophobic guest molecules [8,9]. Depending on the size of the latter, the central apolar cavity of the β-CD molecule can accommodate either an entire lipophilic guest molecule or a sizeable guest residue. On the other hand, the periphery of the toroidally shaped macrocycle features seven hydroxyl groups located on its narrower (primary) rim and fourteen hydroxyl groups on its wider (secondary rim), these functional groups rendering the host–guest complex relatively soluble in water. The use of CDs as vehicles for the delivery of poorly soluble APIs is a major research topic in the fields of supramolecular chemistry and pharmaceutical development. Reports of resulting improvements in drug bioavailability and the enhancement of other pharmaceutically relevant properties abound [10,11,12].

In this report, we describe two distinct crystalline products resulting from the complexation between β-CD and the antifungal API fluconazole (FLU), whose low aqueous solubility limits its efficacy in medicinal applications. This API (trade name Diflucan^®^) is in the same class of azole antifungals as ketoconazole, itraconazole, and miconazole. These drugs are used to treat fungal infections involving the skin and mucous membranes. FLU displays potent fungistatic activity against most strains of Candida microorganisms (e.g., *C. albicans*, *C. glabrata*, *C. parapsilosis*, and *C. tropicalis* [13,14] and is a highly selective inhibitor of lanosterol 14α-demethylase located in the membranes of these microorganisms. This inhibition interrupts the biosynthesis of ergosterol, which is required for fungal cell wall synthesis [13,15]. One of the main advantages of FLU is the option of oral administration (tablets, oral suspension) as well as intravenous injection [16]. The solubility of FLU is often quoted as ≤1 mg/mL, but values in the range 5.2–5.5 mg/mL were reported for various polymorphs of FLU following their isolation by the supercritical antisolvent (SAS) process [17]. This shortcoming of the API and other features of its pharmaceutical profile have been addressed in numerous previous publications aimed at exploring alternative solid forms of FLU that might lead to enhanced drug performance. Isolation of different polymorphs and solvated forms of FLU has been an ongoing activity since the 1990s and some recent studies have focused on different multi-component systems such as co-crystals, two examples of which are highlighted here. In a study by Carneiro et al. [18], four new co-crystals of FLU, namely FLU fumaric acid monohydrate (1:1:1), FLU malic acid (1:1), FLU dipicolinic acid (1:1), and FLU adipic acid (1:1), were synthesized and fully characterized. In 2020, Perlovich et al. [19] reported a comprehensive study of co-crystals of FLU with the aromatic coformer compounds vanillic acid and 4-hydroxybenzoic acid. Both former studies demonstrated that under specified conditions the co-crystals were more soluble than untreated FLU, reflecting the ongoing interest in supramolecular modification of this drug.

Of more significant relevance to the present report, however, are several recent studies that describe attempts to prepare and characterize solid β-CD inclusion complexes of FLU [20,21,22]. The majority of these preparations involved treatments (e.g., kneading, co-evaporation, spray-drying) of β-CD and FLU in a 1:1 molar ratio, an approach that is sometimes based on the results pertaining to their complexation in solution, obtained from phase solubility, nuclear magnetic resonance (NMR), spectroscopic, and fluorescence studies, which indicate 1:1 β-CD FLU complex formation [21]. An exception is a very recent study describing complexation experiments using 1:2, 1:1, and 2:1 molar ratios of β-CD and FLU [23]. However, definitive statements regarding pure complex stoichiometries are generally lacking in these reports. It should be added that no mention of single crystal X-ray diffraction of β-CD complexes of FLU appear in the above reports either and no crystal structures of CD complexes of FLU are currently deposited in the Cambridge Structural Database (CSD) [24].

In this paper, we present unequivocal evidence for the formation of two distinct inclusion complexes with formulae (β-CD)_2_ FLU 27.3H_2_O and (β-CD)_2_ FLU 21.3H_2_O, crystallizing in the triclinic and monoclinic crystal systems, respectively. Following preliminary co-grinding/kneading experiments, these hydrated complexes were isolated in the form of sizeable single crystals via co-precipitation methods and their host–guest stoichiometries were determined by NMR spectroscopy of solutions prepared by dissolving the pure crystalline phases in DMSO-d_6_. Other characterization techniques employed (thermal analysis, X-ray diffraction (XRD) on powders and single crystals, Fourier Transform Infrared (FTIR) spectroscopy) were likewise performed on pure crystalline samples of each complex. Initial rapid identification of these distinct crystalline species as triclinic and monoclinic β-CD complexes was readily achieved using the powder XRD technique and reference patterns for known series of β-CD inclusion complexes. The structures of the complexes were subsequently determined by single crystal XRD, which confirmed that both crystals contain dimeric β-CD units, each host dimer accommodating a single molecule of FLU (ordered in the triclinic phase and disordered over two positions in the monoclinic phase). In addition to the above characterizations, we report a systematic study subsequently carried out to establish the concentration–temperature ranges for optimum isolation of the individual complexes, as well as a description of an experiment illustrating the significant difference in their dehydration rates. Since it is well known, from both previous literature and the references cited above, that β-CD does have the capacity to increase the solubility of FLU, our aim during this study was not to investigate solubility aspects, but rather to gain clarity on the stoichiometry and structures of β-CD complexes of FLU using a more robust methodology than those described in previous publications. Our findings are relevant in the context of reproducible preparation of chemically well-defined CD–FLU complexes and their potential use in drug formulations.

## 2. Results and Discussion

### 2.1. Preliminary Characterization of the Complexes

The triclinic and monoclinic β-CD complexes of fluconazole (TBCDFLU and MBCDFLU, respectively) were prepared independently via co-precipitation methods as described in Section 3.2. Initial powder X-ray diffraction (PXRD) patterns of the two complexes matched the respective reference patterns 12 and 11 of a series of documented isostructural β-CD complexes [25]. These comparisons enabled rapid, unequivocal identification of the respective space groups (P1 and C2) of the β-CD–fluconazole complexes as well as predictions of their approximate unit cell dimensions (viz., lengths of ~15, 15, and 18 Å and angles of ~113, 100, and 102° for TBCDFLU; lengths of ~19, 24, and 16 Å and angles of 90, ~109, and 90° for MBCDFLU). In addition, since the corresponding isostructural β-CD complexes with matching reference PXRD patterns are also known to be based on dimeric host units with distinct dimer complex packing arrangements, it could be deduced that the MBCDFLU complex crystallizes in channel (CH)-type packing while TBCDFLU is based on the “intermediate” (IM)-type packing scheme. The latter packing modes were described in detail earlier [26]. All the preliminary structural features listed above as having been deduced from the experimental PXRD patterns alone were subsequently confirmed by single crystal X-ray analyses of the complexes, as described in Section 2.2.

Determination of the host–guest stoichiometries of the complexes was achieved by dissolving samples of the two crystalline complexes in DMSO-d_6_ and recording the respective ^1^H NMR spectra (Supplementary Materials, Figures S1 and S2 and Tables S1 and S2). Owing to the possibility of mixtures of crystal forms resulting from the co-precipitation experiments, in selecting the samples for NMR analysis to try and ensure phase consistency, reliance was placed on visual inspection to distinguish the different morphologies of crystallites of TBCDFLU, MBCDFLU, and pure β-CD that might also have been present. The phase purities were checked by PXRD prior to NMR analysis. Primary proton signals selected to define the stoichiometric ratios included those of H_1_ of the β-CD molecule and the pair of equivalent protons H_e_ on the triazole rings of the guest FLU. Due to some overlap of signals, perfect integrations were not possible. However, the calculated β-CD:FLU ratio was definitely indicated as being closer to 2:1 than 1:1 for both TBCDFLU and MBCDFLU.

**Figure 2:**
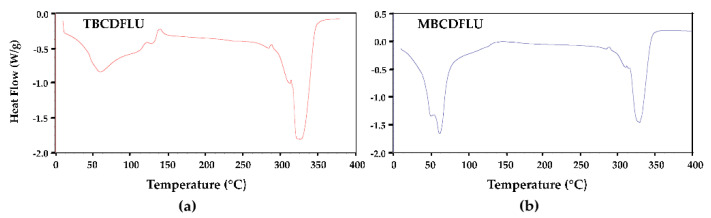
DSC traces for the β-CD fluconazole complexes, (**a**) TBCDFLU and (**b**) MBCDFLU.

β-CD complexes are generally ternary systems, the third component being water, which plays an essential role in maintaining complex crystallinity via multiple hydrogen-bonded networks (e.g., host–water–host, water–water–host, and other combinations). Determination of the water content was thus necessary to fully characterize each complex. Initially, hot stage microscopy (HSM) was used to observe the overall behavior of the two crystal forms on heating single crystal specimens immersed in silicone oil at 10 K min^−1^. HSM micrographs captured in the temperature range 24–320 °C (Supplementary Materials, Figure S3) revealed three thermal events for both crystal forms, namely crystal cracking due to dehydration (a common feature for β-CD complexes), crystal fragmentation, and final decomposition. The differential scanning calorimetry (DSC) trace for TBCDFLU (Figure 2) displayed a single broad endotherm for dehydration with a major peak at ~60 °C, followed by two small endotherms and a small exotherm in the 100–150 °C range.

Instead, the trace for MBCDFLU featured a more intense and narrow endothermic dehydration peak with two distinct components. Above 200 °C, the two DSC profiles are very similar, the resulting anhydrous complexes both displaying a decomposition peak temperature of 325 °C. For each hydrated complex, quantitative determination of water loss on heating was performed using thermogravimetric analysis (TGA). However, this proved to be challenging, especially in the case of the MBCDFLU crystals, which typically dehydrated spontaneously following the manipulations involved in sample preparation for TGA. The routine approach involving multiple measurements on the TGA instrument consequently failed to yield reproducible results. Consistent mass loss data for dehydration were eventually obtained by adding accurately weighed droplets of silicone oil to the rapidly pre-weighed crucible containing surface-dried crystals. Immersion of the crystals in the oil limited their water loss significantly, thereby enabling the TGA runs to be performed routinely thereafter. Further experimental details are provided in Section 3.4. Although the crystals of TBCDFLU did not appear to dehydrate spontaneously at ambient temperature, for uniformity the technique described above was also applied to record their water loss. All TGA data were analyzed as described in Section 3.4. For TBCDFLU, the water content was estimated as 16.7 ± 3.5% (*n* = 11), and the most reliable values yielded 15.0 ± 1.4% (*n* = 3) (Supplementary Materials, Figure S4) corresponding to 25.3 ± 2.4 H_2_O molecules per (β-CD)_2_ FLU complex unit. For MBCDFLU, the estimated water content was 17.9 ± 2.8% (*n* = 4), the most reliable data yielding 16.6 ± 0.8% (*n* = 3) (Supplementary Materials, Figure S4), corresponding to 28.4 ± 1.4 H_2_O molecules per (β-CD)_2_ FLU complex unit. As will be evident in what follows, these experimental estimates of water content are also essential as reference values for modeling the water content in each complex crystal from the respective single crystal X-ray diffraction studies described below.

### 2.2. Crystal Structures of the Complexes

Structure solution and refinement of the two hydrated (β-CD)_2_ FLU complexes presented challenges. In the case of TBCDFLU, persistent twinning of crystal specimens of this species inevitably led to the acceptance of data compromised by this phenomenon. For MBCDFLU, the guest molecule is disordered over two positions satisfying the crystallographic symmetry, which features a twofold rotation axis parallel to the crystal b-axis passing through the centre of the dimeric complex. Additionally, for both crystals some level of disorder of the water molecules was evident. These features therefore involved extensive, sensitive refinements to arrive at acceptable results. The salient crystallographic details are as follows:

Crystal data for TBCDFLU, [(C_42_H_70_O_35_)_2_ (C_13_H_12_F_2_N_6_O) (H_2_O)_27.3_], (M = 3067.56 g/mol): triclinic, space group P1 (no.1), a = 15.331(3) Å, b = 15.392(3) Å, c = 17.972(3) Å, α = 113.613(3)°, β = 99.410(3)°, γ = 102.597(3)°, V = 3640.0(12) Å^3^, Z = 1, T = 173(2) K, μ = 0.128 mm^−1^, D_calc_ = 1.399 g/cm^3^, 13,579 reflections measured (3.0° ≤ 2Θ ≤ 51.4°), 13,579 unique, (R_int_ = 0.0, R_sigma_ = 0.0619), which were used in all calculations. The final R1 was 0.0635 (I > 2σ(I)) and wR2 was 0.1724 (all data).

Crystal data for MBCDFLU, [(C_42_H_70_O_35_)_2_ (C_13_H_12_F_2_N_6_O) (H_2_O)_21.3_], (M = 2959.25 g/mol): monoclinic, space group C2 (no.5), a = 18.879(5) Å, b = 24.408(5) Å, c = 15.375(4) Å, β = 109.862(5)°, V = 6663(3)Å^3^, Z = 2, T = 101(2) K, μ = 0.134 mm^−1^, D_calc_ = 1.475 g/cm^3^, 29,867 reflections measured (2.8° ≤ 2Θ ≤ 55.1°), 15,221 unique, (R_int_ = 0.0361, R_sigma_ = 0.0707), which were used in all calculations. The final R1 was 0.0858 (I > 2σ(I)) and wR2 was 0.2479 (all data).

**Figure 3:**
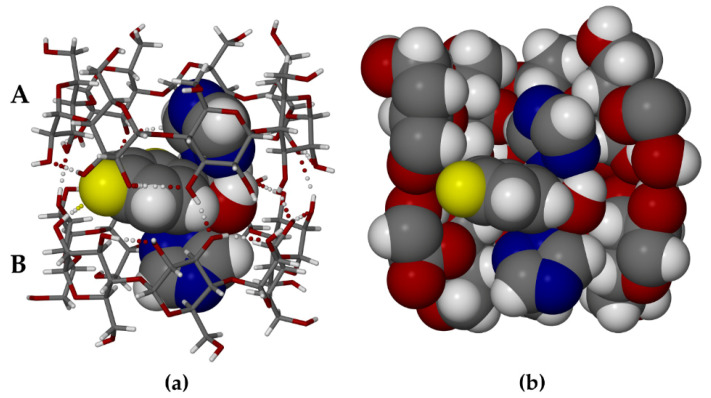
The dimeric complex TBCDFLU: (**a**) the two independent host molecules A and B shown in stick representation with the guest molecule FLU drawn in space-filling mode; (**b**) from the same viewpoint, a cutaway image with host and guest molecules in space-filling style.

The asymmetric unit of TBCDFLU comprises a β-CD dimer, one fluconazole guest molecule, and 27.3 water molecules. Figure 3a is a perspective view of the structure of the TBCDFLU complex (water molecules omitted for clarity).

Within each of the host molecules (**a**) and (**b**) (Figure 3a), contiguous glucose rings (A1–A7 and B1–B7) are generally linked via intramolecular O3 (*n*)—O2 (*n* + 1) hydrogen bonds, while some of the secondary -OH groups also engage in H-bonding with peripheral water molecules. The structure of the well-known β-CD dimer “cage” depicted above is maintained by intermolecular -O-H—O hydrogen bonds between secondary hydroxyl groups on the respective wider secondary rims of the macrocycles. Within this dimeric cage, the FLU molecule adopts a somewhat symmetrical conformation that enables each triazole ring to be completely encapsulated within the hydrophobic cavity of a host molecule, while the bulky difluorophenyl residue and the hydroxyl group are located at the wide interface between the A and B molecules. This appears to be the optimum FLU conformation for its accommodation within the host dimer. Evident is an intramolecular O-H—N hydrogen bond between the FLU hydroxyl group and one of the nitrogen atoms of the triazole ring within the cavity of host molecule A. Resulting close contacts between the FLU molecule and the internal surface of the cage are highlighted in Figure 3b, which confirms the close topological host–guest fit. The specific conformation assumed by the guest molecule upon its inclusion in β-CD is unusual. Of the 29 structural entities in the CSD [24] containing the non-covalently bound FLU molecule (viz., polymorphs, solvates, co-crystals), 27 feature a common ‘asymmetrical’ FLU conformer. Only two entities, one FLU polymorph (IVUQF01) and the FLU 2-hydroxybenzoic acid co-crystal (EZEGIA), contain FLU molecules with similar overall conformations to that in the complex TBCDFLU, but both lack the intramolecular O-H—N hydrogen bond. This reflects some level of adaptation required for the guest molecule to optimize its accommodation within the β-CD dimer. Based on the short intermolecular O2B1-H—F8 distance of ~2.4 Å, there is an indication of a possible hydrogen bond between this secondary -OH group on host B and one of the fluorine atoms (Figure 3a). The fluctional nature of H-bonds involving the hydroxyl groups in cyclodextrin complexes does limit the level of reliability of such details, especially in view of the compromised X-ray data quality from the twinned TBCDFLU crystal. However, it is interesting to note that the corresponding H-bond also occurs in the complex MBCDFLU.

Significant geometrical data defining the β-CD conformations in the TBCDFLU crystal may be derived from the structural results. A key to the labeling of the 14 glucose residues of the β-CD dimer is provided (Supplementary Materials, Figure S5) and the various geometrical parameter values are reported (Supplementary Materials, Table S3). The listed parameters reflecting deviations of the host molecules from regular seven-fold rotational symmetry are defined in the footnote to the table. Here, we mention a few representative data: the ranges for the parameter *l* (the distance between each glycosidic O4 atom and the centroid of the O4-heptagon) are 4.82–5.35 Å for host molecule A and 4.90–5.24 Å for host molecule B; the ranges for Φ (the O4 (*n* − 1)—O4*n*—O4(*n* + 1) angles) are 120.5–134.1° for A and 123.2–132.1° for B; the ranges for τ_2_ (the tilt angle between the mean O4 plane and the mean plane O4-C4…C1-O4′ of each glucopyranose ring) are 4.6–13.7° for A and 4.4–14.8° for B. These ranges and those of the other parameters listed in Table S3 indicate similar magnitudes of host distortion for host molecules A and B, which is consistent with the fact that each accommodates a triazole ring of the fluconazole molecule in a similar fashion.

The refined crystal structure of TBCDFLU was modeled with 18 water oxygen atoms having unit site-occupancy factors (s.o.f.s) and 12 water oxygen atoms with fractional occupancies, the total occupancy being 27.3 in the crystal asymmetric unit (ASU). This value is in reasonable agreement with the estimate of water molecule content per dimeric complex from TGA, namely 25.3 ± 2.4. Water molecules are located at the external surfaces of the β-CD dimer, within hydrogen bonding distances of host oxygen atoms, and they are linked to other water molecules and oxygen atoms of neighboring dimers, leading to a complex network of hydrogen bonds. More detail regarding the important role of water in the reported structures appears below.

**Figure 4:**
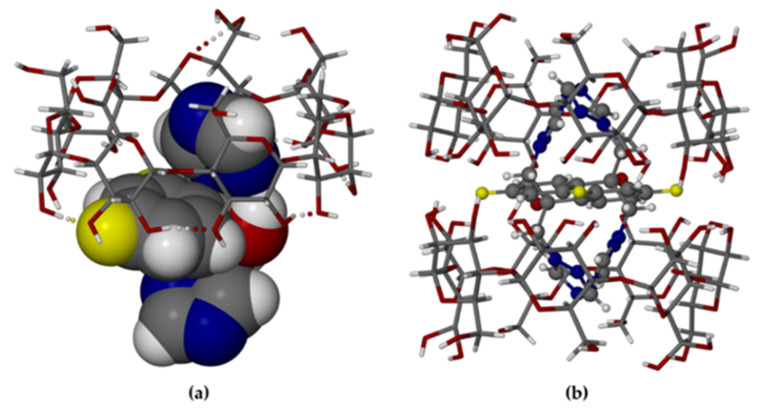
The complex MBCDFLU: (**a**) the asymmetric unit (water oxygen atoms omitted for clarity); (**b**) the (β-CD)_2_ FLU complex unit with disordered FLU components.

MBCDFLU, the second β-CD–fluconazole complex reported here, also comprises a hydrated host dimer that contains one FLU molecule. However, in contrast to TBCDFLU, MBCDFLU crystallizes in the space group C2 with Z = 2, which requires a twofold rotation axis (C_2_) to pass through the dimer interface. As indicated previously, this requirement had been anticipated from the PXRD analysis. The first attempt to solve the structure via isomorphous replacement with the host atomic co-ordinates of a β-CD complex of methylparaben (CSD refcode AJUVEG, [27]) was based on intensity data collected at 173 K. While the host structure refined successfully and water oxygens could be placed, the electron density within the β-CD cavity was extremely low and uninterpretable, preventing any form of guest modeling. The results reported here, based on a subsequent data collection performed at 100 K, enabled both the location of the disordered guest atoms from successive difference Fourier syntheses and their satisfactory refinement. The asymmetric unit (ASU) in the modeled crystal structure of MBCDFLU (Figure 4a) comprises one β-CD molecule, one-half of a FLU molecule (i.e., s.o.f. = 0.5), and 10.6 water oxygen atoms.

**Figure 5:**
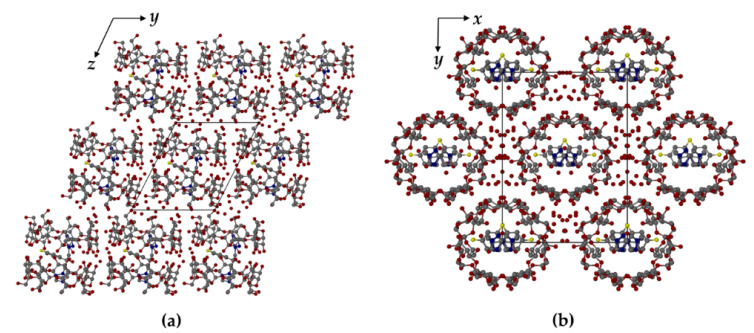
Crystal packing arrangements in TBCDFLU, (100) projection (**a**) and MBCDFLU, (001) projection (**b**). For clarity, no H atoms are included. Water oxygen atoms are shown as isolated red spheres. The C-centered arrangement of the complex units in (**b**) is evident.

Applying the C_2_ operation to the ASU (Figure 4a) produces the dimeric (β-CD)_2_ FLU complex structure (Figure 4b), with the complete formula for the hydrated complex quoted in the crystallographic data listed above. Both components of the disordered FLU molecule are shown in Figure 4b, which is a view along the C_2_ axis passing through the common atom F7 (central yellow sphere) located at the special position ½, y = 0.3989(3), ½. A more detailed view of the disordered guest is provided in the Supplementary Materials as Figure S6. While the FLU molecule is disordered over two geometrically equivalent positions, the mode of its inclusion is analogous to that found in TBCDFLU, namely accommodation of each triazole ring within the cavity of a β-CD molecule with the difluorophenyl and hydroxyl groups located at the dimer interface. As such, it is expected that the ranges of the geometrical parameters describing the host conformation in MBCDFLU (Supplementary Materials, Figure S7 and Table S4) should be very similar to those summarized above for TBCDFLU. This is confirmed by the following parameter ranges observed for MBCDFLU, namely *l*: 4.83–5.29 Å; Φ: 122.1–131.3°; τ_2_: 2.5–14.4°. We note that for the included FLU molecule in MBCDFLU, the same pair of H-bonds observed in the TBCDFLU structure, namely the intramolecular -OH–N H-bond in FLU and the intermolecular (secondary)-OH–F H-bond, also occur in this complex (Figure 4a). Regarding the water content of MBCDFLU, which dehydrated rapidly at ambient conditions, it has already been mentioned that the rigorous non-routine TGA technique used to quantify it using a bulk sample of the complex in fact yielded a mass loss corresponding to 28.4 ± 1.4 water molecules per (β-CD)_2_ FLU complex unit. However, from the single crystal X-ray data, a tally of only 21.3 water oxygen atoms was recorded. This discrepancy is attributed to spontaneous loss of some water content from the single crystal during manipulations involved in the lengthy (20 h) intensity data collection. The water molecules engage in complex hydrogen-bonded networks in both crystal forms (Figure 5), details of which can be gauged from the H-bond tables (files with extension sup in Supplementary Materials) and short O—O contacts.

In TBCDFLU, the complex units are arranged in the IM (“intermediate”) packing type [25,26] (Figure 5a), characterized by a lateral shift of successive complex layers, such that the primary sides of each dimer are blocked by two neighboring dimeric complexes in the layers above and below. Instead, the complex units in MBCDFLU are assembled in CH (“channel”) packing mode [25,26]. Figure 5b shows the view parallel to the channels.

### 2.3. Complex Dehydration

The specific packing arrangements depicted in Figure 5 were discussed in a previous communication [27] in the context of the dehydration features for two 1:1 β-CD complexes of methylparaben (MPB) crystallizing in the respective IM and CH arrangements. These β-CD MPB complexes displayed analogous behaviour to those described in the present report, the complex with the IM packing type being relatively resistant to dehydration at ambient temperature and that with the CH packing type demonstrating rapid spontaneous dehydration. In the account of the methylparaben complexes, a plausible explanation for the relative stability of the IM packing type towards dehydration was based on the location of relatively low concentrations of water molecules within interstices surrounding the close-packed complex dimer units; instead, in the CH packing type, the infinite columns of aligned complex dimers are separated by high concentrations of water molecules located in linear channels parallel to the columns, facilitating their diffusion out of the crystal.

In the present study, an investigation of the relative speeds of dehydration of TBCDFLU and MBCDFLU crystals was performed, the results of which are presented in a series of successive micrographs captured over a period of 3 min (Supplementary Materials, Figure S8). Single crystals of the two complexes in a thin layer of their mother liquor were placed on a microscope slide. It was observed that, following complete evaporation of the mother liquor (“time zero”), within 12 s the MBCDFLU crystal had begun to darken with commencement of cracking due to dehydration. After a total of 18 s, the crystal was opaque and remained in that condition. Instead, the TBCDFLU crystal suffered minimal cracking and was still transparent after ~3 min.

### 2.4. Further Solid-State Characterization by FTIR and PXRD

Recording of the FTIR spectra of TBCDFLU and MBCDFLU was performed as part of their characterization. These spectra showed no significant differences (Supplementary Materials, Figure S9). This is not surprising given the common complex chemical formula (β-CD_2_ FLU) and similar water contents in the two crystal forms. In addition, characteristic peaks of FLU in the spectra are dwarfed by those of the host β-CD due to the relative mass ratio of 1:7.4 for these components. FTIR spectroscopy is therefore not effective for discriminating the two complexes. Instead, the distinct PXRD patterns of TBCDFLU and MBCDFLU enable rapid identification of the two forms. Furthermore, the application of PXRD in this context is essential for demonstrating that the patterns obtained from the bulk samples of the complexes are in accord with the respective patterns calculated from the single crystal X-ray structure determinations. This requirement is duly satisfied and the relevant PXRD patterns are provided (Supplementary Materials, Figure S10).

### 2.5. Isolation of the Individual Crystal Forms

Finally, to provide guidance for the isolation of pure samples of TBCDFLU and MBCDFLU, a detailed study of the crystallization conditions was performed. Essentially, it was determined that if a co-precipitation procedure was used, the product(s) of crystallization (pure TBCDFLU, pure MBCDFLU, or mixtures of the two crystal forms) depended on two variables, namely the incubation temperature of solutions containing β-CD and FLU in a 2:1 molar ratio, and the solute concentration (expressed as the molar concentration of β-CD) (Supplementary Materials, Table S5). Pure MBCDFLU could be isolated at a β-CD concentration of 6.5 × 10^−2^ M when the incubation temperature was maintained at 60 °C, while pure TBCDFLU could be isolated at the same β-CD concentration but only if the incubation temperature was ≤45 °C. Other conditions generally produced a mixture of the two crystal forms. Further experimental details are provided in Section 3.2. Since a popular method of complex preparation involves kneading the host and guest with water present, this method was also explored, with the finding that pure TBCDFLU could be produced by kneading a 2:1 mixture of β-CD and FLU. When this product was recrystallized and the solution then incubated at 60 °C, the final crystallization yielded MBCDFLU.

## 3. Materials and Methods

### 3.1. Materials

β-cyclodextrin (β-CD; C_42_H_70_O_35_) with purity > 95% (code CY-2001) was purchased from Cyclolab, Budapest, Hungary. Fluconazole (PHR1160) was obtained from Sigma-Aldrich, Kempton Park, South Africa. Both materials were used as received.

### 3.2. Optimization of Individual Complex Crystal Form Isolation

Reproducible preparation of single crystals of TBCDFLU and MBCDFLU by co-precipitation experiments was optimized by observing the crystallization outcomes accompanying both the variation in incubation temperature of aqueous solutions containing β-CD and FLU in a 2:1 stoichiometric ratio and the solution concentrations. The procedure involved initial preparation of a solution of β-CD (37 mg, 0.032 mmol) in 0.5 mL of pure water. The solution was heated to 60 °C with constant stirring and a total of 5 mg (0.016 mmol) of FLU was added at the rate of 1 mg per h. Stirring continued for 20–24 h. The hot solution was then rapidly filtered (0.45 μm nylon filter) into a vial immersed in a Dewar flask containing water at 60 °C. The solution was thermally isolated and left to incubate by very slow cooling over two days, when large colorless crystals appeared. It was established that maintaining the initial solution temperature of 60 °C led to the monoclinic form MBCDFLU (M) exclusively, whereas if the procedure commenced with the solution at 45 °C, only crystals of TBCDFLU (T) were obtained. Further experiments at each of the above temperatures followed, with solutions obtained by serial dilution of the initial β-CD/FLU solution, the crystallization outcomes revealing either mixtures of M and T, or form T alone. Crystal form identification was determined by random selection of three crystals from each crystallization batch and measurement of their unit cell dimensions on the diffractometer. PXRD was subsequently used to check the homogeneity of the remaining crystals in each vial. A manual kneading experiment (~15 min) was performed with the same masses of the two components listed above, with the addition of ~20 μL of water.

### 3.3. Host–Guest Stoichiometry Determination

Crystals of TBCDFLU and MBCDFLU were dissolved in DMSO-d_6_ and their ^1^H NMR spectra were recorded on a Bruker 300 MHz spectrometer. Host–guest stoichiometric ratios were based on peak integrations.

### 3.4. Thermal Analysis

Hot stage microscopy (HSM) was performed with a Linkam THMS600 instrument (Linkam Scientific Instruments, Tadworth, UK) fitted with a TP92 temperature control unit. Samples were generally covered by a thin layer of silicone oil on a microscope slide and their behavior on heating was viewed with a Nikon SMZ-10 stereomicroscope (Tokyo, Japan). A Sony Digital Hyper HAD video camera was used to record images. The Soft Imaging System program analySIS was used for image processing. Thermogravimetric analysis (TGA) was performed with a TA-Q500 instrument (TA Instruments, New Castle, DE, USA) using Universal Analyzer software (v4.5A, TA Instruments-Waters LLC, New Castle, DE, United States). Operating conditions included a heating rate of 10 K min^−1^ and a dry nitrogen purge gas flow rate of 60 cm^3^ min^−^^1^. Due to the rapid (1–2 min) dehydration of crystals of MBCDFLU and consequent variability in estimates of its water content via routine TGA, a procedure involving immersion of complex crystals in silicone oil for TGA was devised. Crystal samples were removed from their mother liquor, rapidly dried on filter paper, and placed in a pre-weighed crucible. The loaded crucible was then weighed in a few seconds and the silicone oil added before the final weighing, which was followed by rapid placement of the crucible on the TG apparatus for analysis. While each of the samples of MBCDFLU weighed ~5 mg, addition of silicone oil resulted in final masses of ~40 mg. The TGA curves were corrected for the extra mass due to the additions of oil and this resulted in significantly more consistent results for the percentage mass loss due to dehydration. In the interest of uniformity, the same procedure was used to quantify the water content of TBCDFLU crystals. Despite every effort to obtain highly consistent results, considerable scatter in the percentages for water loss was evident since the required manipulations involving addition of silicone oil were very demanding and not always optimally performed. For TBCDFLU, fifteen TGA traces were recorded, of which 11 involved the addition of silicone oil. For all 11 data, including outliers, the water content was 16.7 ± 3.5%, while the three most reliable values yielded 15.0 ± 1.4%. For MBCDFLU, ten TGA traces were recorded, four of them involving the addition of silicone oil. For all four measurements, the water content was estimated at 17.9 ± 2.8%, while the three most reliable data yielded 16.6 ± 0.8%. For differential scanning calorimetry (DSC), a DSC25 instrument (TA Instruments, New Castle, DE, USA) using TRIOS software (v4.1.0.3179, TA Instruments-Waters LLC) was employed. Surface-dried crystals with masses in the range 1.5–3.0 mg were placed in vented aluminum pans and the dry nitrogen purge gas flow rate was 60 cm^3^ min^−1^.

### 3.5. Single Crystal X-ray Diffraction Analysis

Reflection intensities from crystal specimens were measured on a Bruker Kappa Apex II Duo diffractometer (Madison, WI, USA) using MoKα X-rays, with the crystals mounted on nylon loops with Paratone N oil (Exxon, Chemical Co., TX, USA) and cooled in a nitrogen vapor stream from a cryostream cooler (Oxford Cryosystems Ltd., Oxford, UK). The paucity of adequately sized single crystals of TBCDFLU necessitated the choice of a twinned specimen, while crystal twinning was not evident for MBCDFLU. Intensity data were corrected for Lorentz polarization and absorption effects (Supplementary Materials, CIF files also listing all program details). The structures of TBCDFLU and MBCDFLU were solved by isomorphous replacement, the respective trial models being the atomic co-ordinates of the glucose rings of the β-CD molecules in the asymmetric units of a geraniol complex (CSD refcode VUYGUT, space group P1 [24]) and a methylparaben complex (refcode AJUVEG, space group C2 [24]). The fluconazole and water molecules were subsequently located in successive difference Fourier maps. To obtain acceptable models, extensive least-squares refinements were required in each case due to the compromised data for TBCDFLU (refined as a two-component twin with major fractional contribution 0.77) and the disordered FLU molecule in the MBCDFLU structure. Non-hydrogen atoms were generally refined anisotropically, except in cases where this was not warranted (e.g., FLU atoms with half-occupancy, or where anisotropic treatment was unstable). For MBCDFLU, 18 distance restraints were imposed to maintain geometries based on initial electron-density peaks and an EADP restraint was imposed on one of the disordered triazole rings. Hydrogen atoms were generally placed in idealized positions and in H-bonding positions for hydroxyl H atoms. No hydrogen atoms were assigned to water oxygen atoms due to their general absence in difference Fourier syntheses (a typical situation for β-CD complexes). Water oxygen atoms were located on 30 sites in TBCDFLU, the sum of the site-occupancy factors (s.o.f.s) corresponding to 27.3 water molecules per (β-CD)_2_ FLU unit. For the asymmetric unit of MBCDFLU, there were 22 unique water oxygen atom sites, the sum of the s.o.f.s corresponding to 21.3 water molecules per (β-CD)_2_ FLU unit.

### 3.6. Powder X-ray Diffraction (PXRD) Analysis

PXRD patterns were recorded on a D8 Advance X-ray diffractometer (Bruker, Karlsruhe, Germany) using CuKα_1_-radiation (λ = 1.5406 Å). Powder samples were mounted on a zero-background holder rotating at 10 rpm. The scanning range was 4.0–40.0° with a step size of 0.05° per second. X-rays were generated with settings 30 kV and 40 mA.

### 3.7. Fourier Transform Infrared (FTIR) Spectroscopy

Instruments for recording infrared spectra included a Bruker Tensor 27 spectrometer (Bruker Optik GmbH, Ettlingen, Germany) equipped with an attenuated total reflectance (ATR) platinum Diamond 1 accessory for solid samples, and a PerkinElmer 100 FT-IR instrument (Shelton, CT, USA) fitted with a Universal Attenuated Total Reflectance (UATR) Accessory. Spectra in the range 400–4000 cm^−1^ were recorded.

## 4. Conclusions

To a large extent, recent literature reports on the solid-state interaction between β-CD and the antifungal drug fluconazole (FLU) have been based on preparations using equimolar quantities of the two components [20,21,22], in some cases with serious shortcomings in the interpretation of the results. In particular, in most cases, if complexation apparently occurred, either a 1:1 inclusion complex was assumed to have been produced, or no further discussion of complex stoichiometry followed. This does not rule out possible formation of a 1:1 complex in the solid state. However, if the true stoichiometry of the resultant inclusion complex was, e.g., 2:1 and all the β-CD formed the complex, the mixed product would contain an excess of the guest FLU, and since the complex would dominate in the mixture, the PXRD pattern (for example) of this mixture would be deceptive, leading to the false conclusion of 1:1 complexation. While initial attempts to prepare complexes between CDs and guest compounds may indeed generally commence with equimolar amounts of host and guest, subsequent application of thermal, spectroscopic, and X-ray diffraction methods is essential to determine the true stoichiometry of any complex product. In this regard, attempted preparation using not only a 1:1 host–guest molar ratio, but also e.g., 2:1 and 1:2, is a more reliable procedure to adopt [23] and could potentially also reveal the existence of more than one specific complex product between the host and guest in question.

In the present case, the combination of crystallization studies and the characterization methods listed above has not only eliminated ambiguity regarding complex stoichiometry under the conditions employed but has also resulted in the rare instance of isolation of two crystallographically distinct complexes, namely the 2:1 hydrated (β-CD)_2_ FLU complexes reported in this paper. Precise conditions for their isolation in phase-pure forms have also been described. Furthermore, their significantly different rates of dehydration are of practical relevance in the context of choosing a suitable candidate complex for possible further manipulation in pharmaceutical development.

## Data Availability

Additional Supplementary Data for TBCDFLU and MBCDFLU (.sup files); CIF files: CCDC 2092248 (TBCDFLU) and 2092251 (MBCDFLU) contain the supplementary crystallographic data for this paper. These data can be obtained free of charge via http://www.ccdc.cam.ac.uk/conts/retrieving.html (or from the CCDC, 12 Union Road, Cambridge CB2 1EZ, UK; Fax: +44-1223-336033; E-mail: deposit@ccdc.cam.ac.uk).

## Supplementary Materials

The following are available online: Figure S1. ^1^H NMR spectrum of the TBCDFLU complex (solvent DMSO-d_6_); Table S1. ^1^H NMR spectral integration for the TBCDFLU complex; Figure S2. ^1^H NMR spectrum of the MBCDFLU complex (solvent DMSO-d_6_); Table S2. ^1^H NMR spectral integration for the MBCDFLU complex; Figure S3. HSM micrographs showing the behavior of hydrated β-CD FLU complexes on heating under silicone oil at 10 K min^−1^; Figure S4. Magnified views of selected TGA curves for the dehydration of TBCDFLU and MBCDFLU; Figure S5. Perspective view of the host molecules A and B in TBCDFLU (H atoms omitted for clarity) showing the labeling of the glucose residues; Table S3. Geometrical parameters of the host molecules (A, B) in TBCDFLU; Figure S6. Atomic numbering of the fluconazole molecule in the MBCDFLU crystal (left) and a general view of the two disordered guest components (right); Figure S7. Atomic numbering of the glucose residues in the β-CD molecule of MBCDFLU; Table S4. Geometrical parameters of the host molecule MBCDFLU; Figure S8. Relative dehydration rates of single crystals of TBCDFLU (T) and MBCDFLU (M), with times in seconds indicated on the micrographs; Figure S9. FTIR spectra for (top) the host (β-CD), the guest (fluconazole), and the co-precipitated product TBCDFLU, and (bottom) the co-precipitated product MBCDFLU; Figure S10. Experimental and calculated PXRD patterns for TBCDFLU and MBCDFLU; Table S5. Experimental conditions for the preparation of crystal forms TBCDFLU and MBCDFLU.

## Sample Availability

Not available from the authors.
