# Supplementary material for: Two Crystal Forms of a Hydrated 2:1 β-Cyclodextrin Fluconazole Complex: Single Crystal X-ray Structures, Dehydration Profiles, and Conditions for Their Individual Isolation
Source: Molecules. 2021 Jul 22;26(15):4427. doi: 10.3390/molecules26154427 (PMC8348925; doi:10.3390/molecules26154427)
Supplement: Supplementary file 1 [file molecules-26-04427-s001.zip › molecules-1297359-supplementary.pdf]

Two crystal forms of a hydrated 2:1 β-cyclodextrin·fluconazole complex: single crystal X-ray structures, dehydration profiles, and conditions for their individual isolation

Andrea Sala, Zakiena Hoossen, Alessia Bacchi, and Mino R. Caira

SUPPLEMENTARY MATERIAL

Figure S1. ^{1}H NMR spectrum of the TBCDFLU complex (solvent DMSO-d_6).

Table S1. ^{1}H NMR spectral integration for the TBCDFLU complex.

Figure S2. ^{1}H NMR spectrum of the MBCDFLU complex (solvent DMSO-d_6).

Table S2. ^{1}H NMR spectral integration for the MBCDFLU complex.

Figure S3. HSM micrographs showing the behavior of hydrated β-CD·FLU complexes on heating under silicone oil at 10 K min^{-1}.

Figure S4. Magnified views of selected TGA curves for the dehydration of TBCDFLU and MBCDFLU.

Figure S5. Perspective view of the host molecules A and B in TBCDFLU (H atoms omitted for clarity) showing the labeling of the glucose residues.

Table S3. Geometrical parameters of the host molecules (A, B) in TBCDFLU.

Figure S6. Atomic numbering of the fluconazole molecule in the MBCDFLU crystal (left) and a general view of the two disordered guest components (right).

Figure S7. Atomic numbering of the glucose residues in the β-CD molecule of MBCDFLU.

Table S4. Geometrical parameters of the host molecule MBCDFLU.

Figure S8. Relative dehydration rates of single crystals of TBCDFLU (T) and MBCDFLU (M), with times in seconds indicated on the micrographs.

Figure S9. FTIR spectra for (top) the host (β-CD), the guest (fluconazole), and the co-precipitated product TBCDFLU, and (bottom) the co-precipitated product MBCDFLU.

Figure S10. Experimental and calculated PXRD patterns for TBCDFLU and MBCDFLU.

Table S5. Experimental conditions for the preparation of crystal forms TBCDFLU and MBCDFLU.

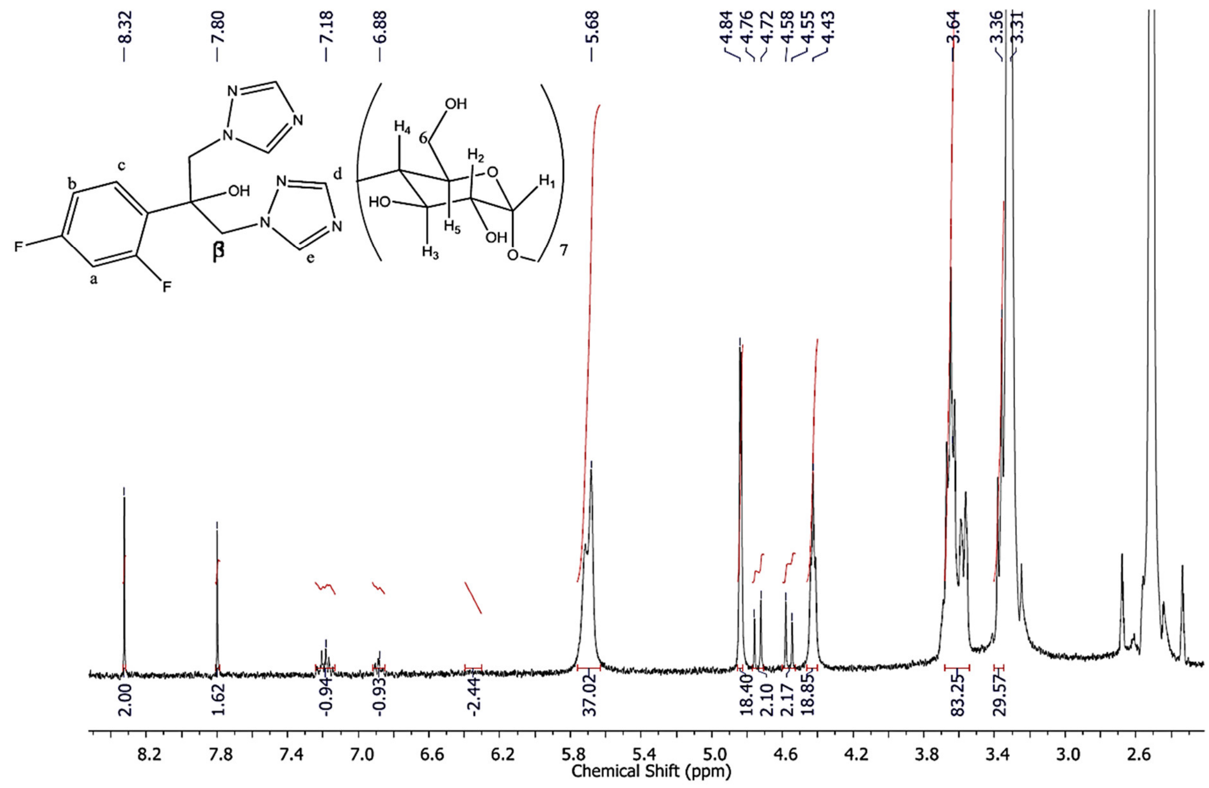

Figure S1. ^{1}H NMR spectrum of the TBCDFLU complex (solvent DMSO-d$_6$).

Table S1. ^{1}H NMR spectral integration for the TBCDFLU complex.

Proton	δ(ppm)	Multiplicity	J(Hz)	Int	Experimental/ theoretical
C-H_e	8.33	s		*2.00	1.00
C-H_d	7.80	s		1.62	0.81
C-H_b	6.88	m		0.93	0.93
C-O$\underline{H}_2$ & C-O$\underline{H}_3$	5.72 & 5.69	2×s		37.02	1.32
C-$\underline{H}_1$	4.84	s		18.40	1.31
C-$H_{β1}$	4.72	d	14.6	2.10	1.05
C-$H_{β2}$	4.54	d	14.3	2.17	1.09
C-O$\underline{H}_6$	4.43	s		18.85	1.35

*Reference integral

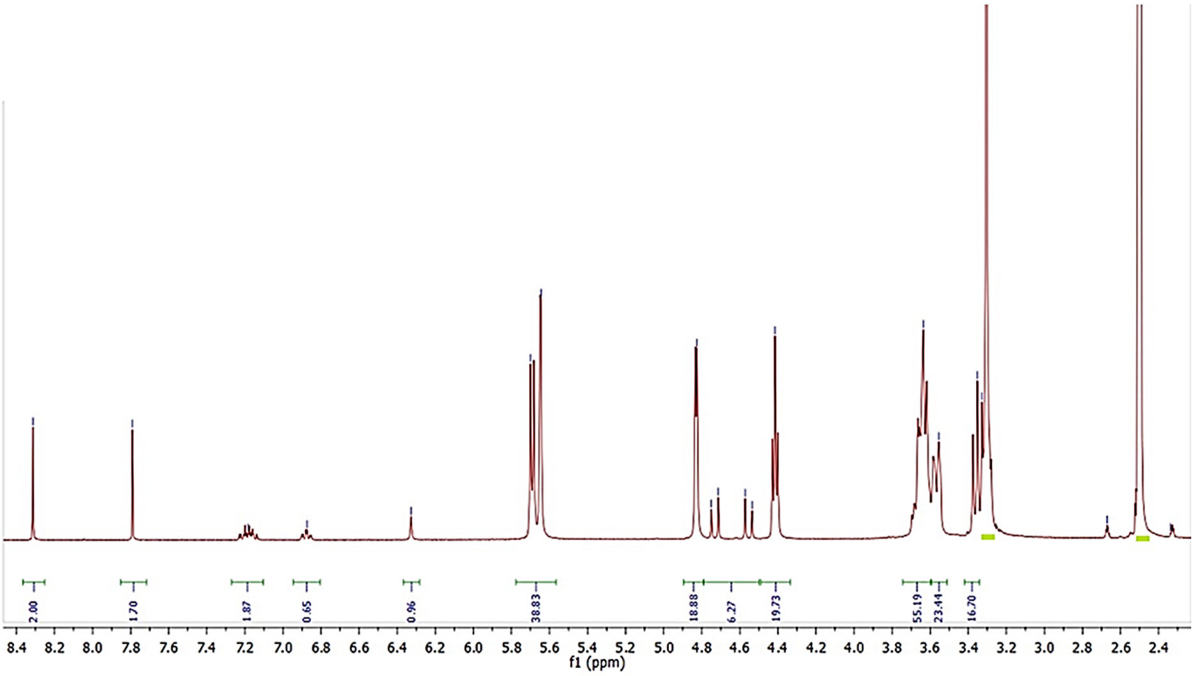

Figure S2. ^{1}H NMR spectrum of the MBCDFLU complex (solvent DMSO-d$_6$).

Table S2. ^{1}H NMR spectral integration for the MBCDFLU complex.

Proton	δ (ppm)	Multiplicity	J/Hz	Int	Experimental/ theoretical
C-H$_e$	8.31-8.32	s		*2.00	1.00
C-H$_d$	7.79	s		1.70	0.85
C-H$_{b,c}$	7.12-7.24	m		1.87	0.96
C-H$_a$	6.84-6.92	d of t	1.8, 6	0.65	0.65
O-H$_d$	6.32-6.33	s		0.96	0.96
C-O$\underline{H_2}$ & C-O$\underline{H_3}$	5.58-5.74	d x 2	5.1, 1.8	38.83	1.39
C-$\underline{H}_1$	4.79-4.87	d	2.7	18.88	1.34
C-$\underline{H}_{\beta 1}$ & C$\underline{H}_{\beta 2}$	4.50-4.78	q	12, 54	6.27	1.56
C-O$\underline{H}_6$	4.35-4.46	s		19.73	1.57

*Reference integral

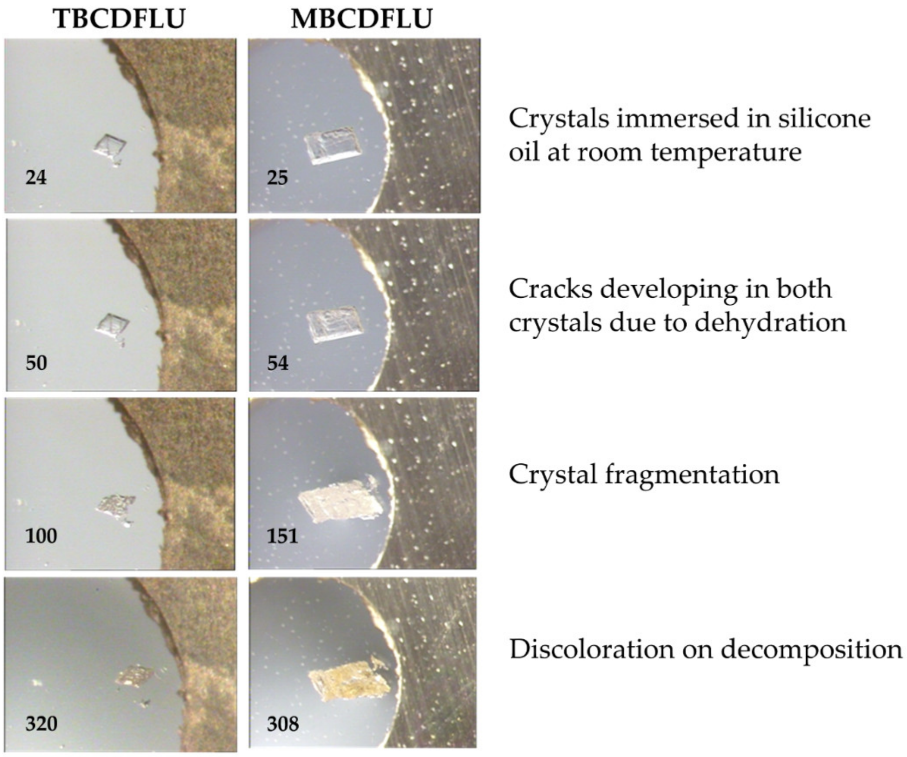

Figure S3. HSM micrographs showing the behavior of hydrated β-CD·FLU complexes on heating under silicone oil at 10 K min^{-1}.

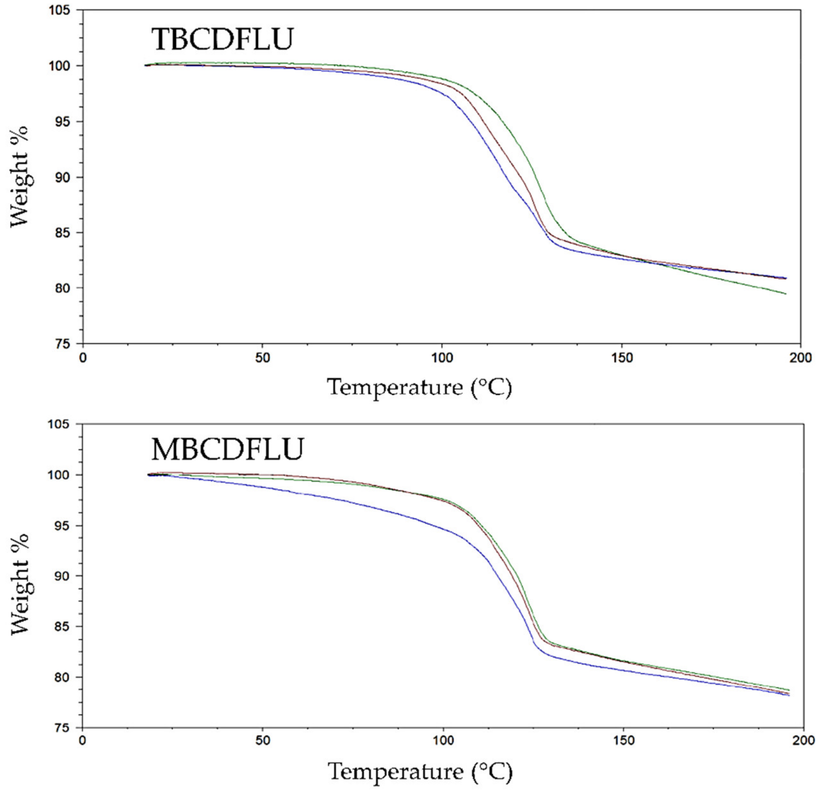

Figure S4. Magnified views of selected TGA curves for the dehydration of TBCDFLU and MBCDFLU.

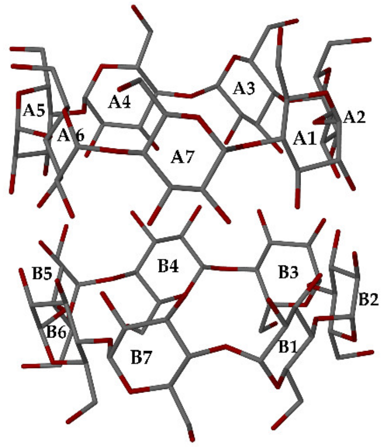

Figure S5. Perspective view of the host molecules A and B in TBCDFLU (H atoms omitted for clarity) showing the labeling of the glucose residues. (Geometrical data tabulated below).

Table S3. Geometrical parameters of the host molecules (A, B) in TBCDFLU.

Residue	*l* (Å)	D (Å)	Φ (°)	d	α[a] (Å)	D_3[b] (Å)	τ_2[c] (°)
A1	5.012	4.533	132.8	-0.4	0.015	2.85	8.9
A2	4.821	4.288	126.8	-0.3	0.007	2.79	4.6
A3	5.121	4.418	124.8	1.6	-0.013	2.85	6.8
A4	5.226	4.247	130.7	-1.9	-0.011	2.85	11.7
A5	4.904	4.560	134.1	0.5	0.030	2.81	9.5
A6	4.847	4.303	120.5	0.5	-0.017	2.70	13.7
A7	5.352	4.293	130.2	-0.3	-0.011	2.81	7.7
B1	5.025	4.392	130.1	-0.9	0.000	2.87	13.8
B2	4.900	4.404	129.8	-0.3	0.014	2.71	4.4
B3	5.007	4.381	123.2	2.2	-0.014	2.76	8.5
B4	5.239	4.203	132.1	-3.0	-0.013	2.79	8.4
B5	4.899	4.570	131.0	1.9	0.037	2.83	8.7
B6	4.903	4.263	124.1	-0.9	-0.029	2.83	14.8
B7	5.211	4.319	129.6	0.9	0.006	2.80	6.3

[a] mean e.s.d. 0.004 Å; [b] mean e.s.d. 0.01 Å; [c] mean e.s.d. 0.2°

PARAMETER DEFINITIONS

***l*:** distance between each glycosidic O4 atom and the centroid of the O4-heptagon.

D: glycosidic O4*n*···O4(*n*+1) distance.

Φ: O4(*n*-1)···O4*n*···O4(*n*+1) angle.

d: O4(*n*-1) ··O4*n*···O4(*n*+1)···O4(*n*+2) torsion angle.

α: deviation of atoms O4*n* from the least-squares plane through the O4-heptagon.

D_3: inter-ring hydrogen bond O(2*n*)···O(3*n*-1) distances.

τ_2: tilt angle (between O4n-C1n…C4n-O4(n+1) mean plane and that of the O4-heptagon).

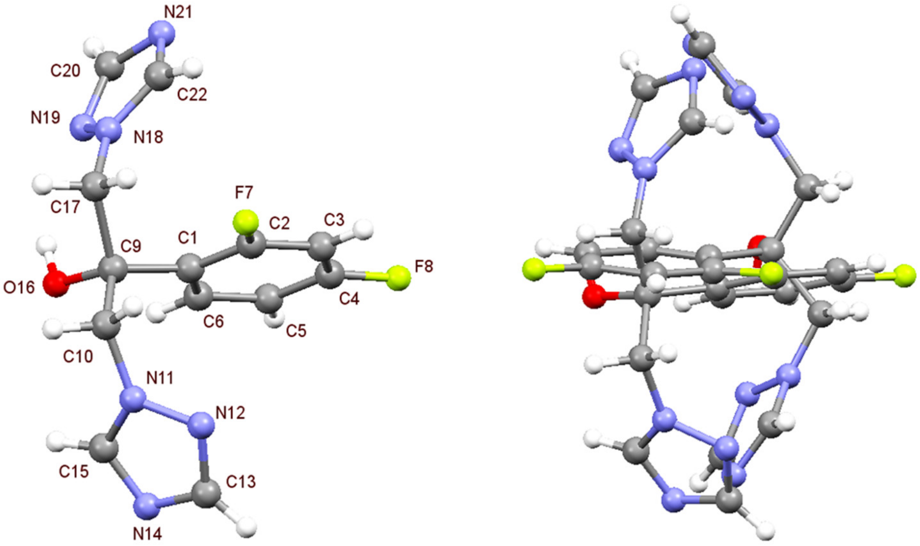

Figure S6. Atomic numbering of the fluconazole molecule in the MBCDFLU crystal (left) and a general view of the two disordered guest components (right).

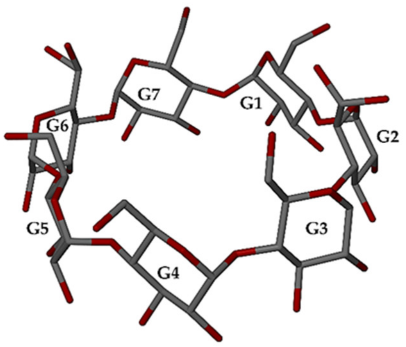

Figure S7. Atomic numbering of the glucose residues in the β-CD molecule of MBCDFLU. (Geometrical data tabulated below).

Table S4. Geometrical parameters of the host molecule MBCDFLU.

**Residue**	***l* (Å)**	**D (Å)**	**Φ (°)**	**d (°)**	**α[a] (Å)**	**D_3[b] (Å)**	**τ_2[c] (°)**
G1	4.878	4.381	130.6	-3.4	-0.071	2.830	11.3
G2	5.097	4.311	127.8	1.7	-0.041	2.788	13.1
G3	5.194	4.275	124,5	4.5	0.090	2.801	10.3
G4	4.833	4.534	130.4	-5.6	0.011	2.786	2.5
G5	4.951	4.294	130.4	0.4	-0.115	2.763	10.6
G6	5.292	4.337	122.1	3.6	0.062	2.787	14,4
G7	4.956	4.436	131.3	-0.7	0.064	2.961	9.3

[a] mean e.s.d. 0.004 Å; [b] mean e.s.d. 0.008 Å; [c] mean e.s.d. 0.2°

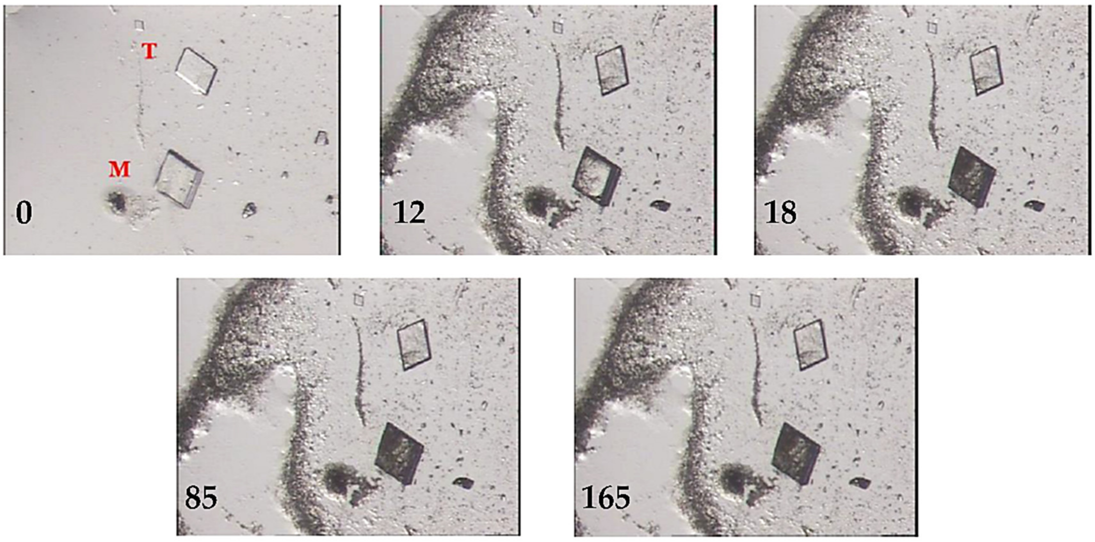

Figure S8. Relative dehydration rates of single crystals of TBCDFLU (T) and MBCDFLU (M), with times in seconds indicated on the micrographs.

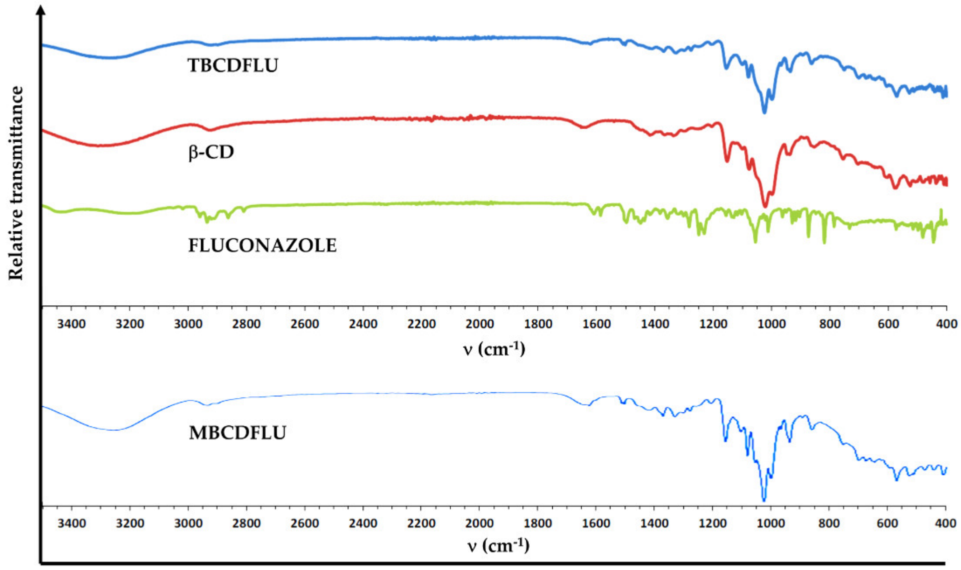

Figure S9. FTIR spectra for (top) the host (β-CD), the guest (fluconazole), and the co-precipitated product TBCDFLU, and (bottom) the co-precipitated product MBCDFLU.

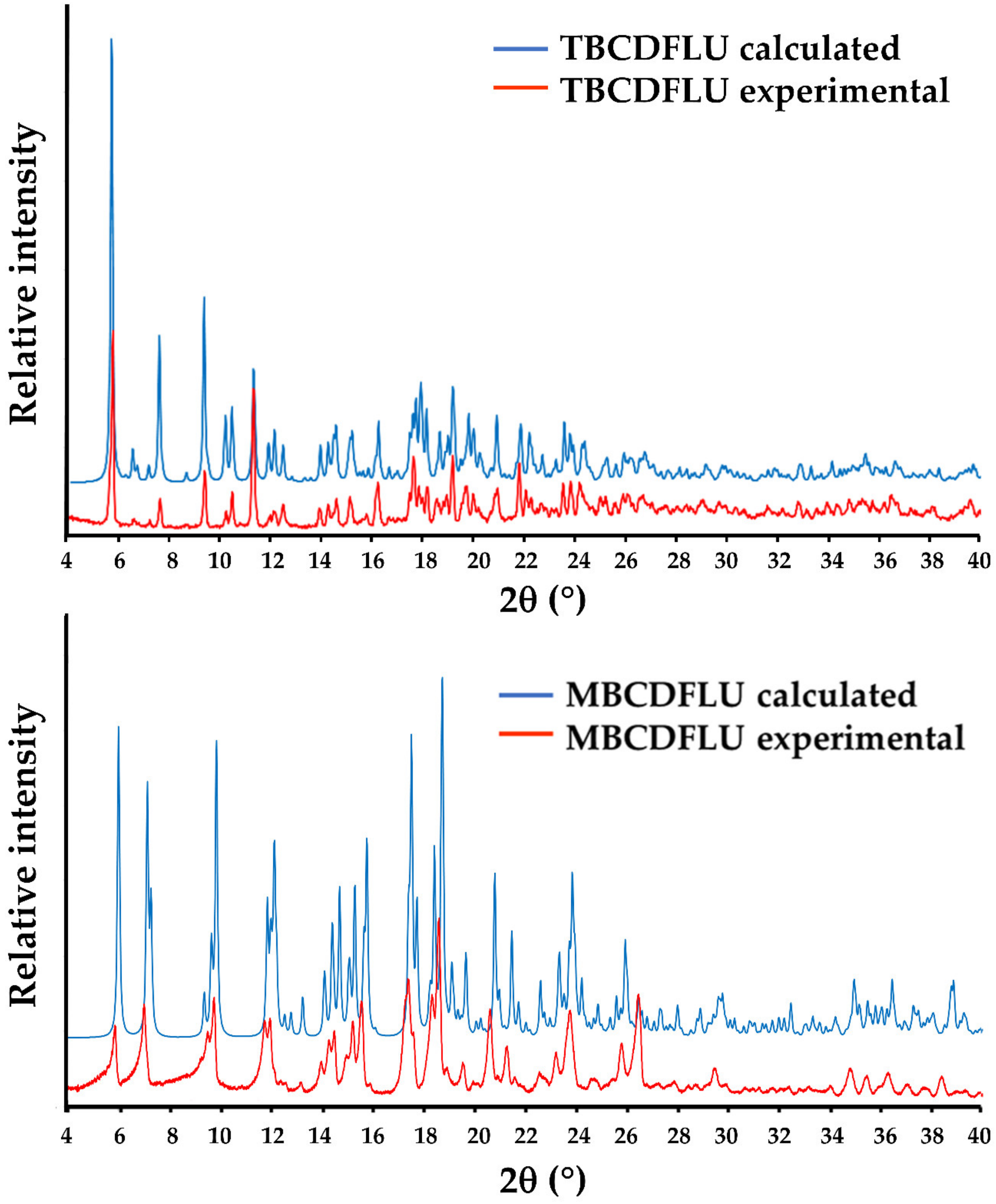

Figure S10. Experimental and calculated PXRD patterns for TBCDFLU and MBCDFLU. The shifts of the majority of the peaks in the calculated pattern of MBCDFLU to slightly higher 2θ-positions in the figure are due to the large difference in the temperatures of the recording of the experimental pattern (294 K) and the calculated pattern based on the single-crystal X-ray analysis (100 K).

Table S5. Experimental conditions for the preparation of crystal forms TBCDFLU and MBCDFLU.

Name	V H_2O (ml)	mg Fluconazole	mg β-CD	Concentration (M)	Incubation T.	Crystals	Type of form
50-4 AS	0.5	5	37	6.52×10^{-2}	60 °C	√	**M**
SET No. 1	0.5	4	29.6	5.21×10^{-2}	60 °C	√	**M + T**
	0.5	3	22.2	3.91×10^{-2}	60 °C	√	**M + T**
	0.5	2	14.8	2.61×10^{-2}	60 °C	√	**M + T**
	0.5	1	7.4	1.3×10^{-2}	60 °C	√	**T**
	0.5	0.5	3.7	6.52×10^{-3}	60 °C	×	×
50-10 AS	0.5	5	37	6.52×10^{-2}	45 °C	√	**T**
SET No.2	0.5	4	29.6	5.21×10^{-2}	45 °C	√	**M + T**
	0.5	3	22.2	3.91×10^{-2}	45 °C	√	**T**
	0.5	2	14.8	2.61×10^{-2}	45 °C	√	**T**
	0.5	1	7.4	1.3×10^{-2}	45 °C	√	**T**
	0.5	0.5	3.7	6.52×10^{-3}	45 °C	×	×

*The designations 50-4 AS and 50-10-AS are in-house code names for the specific conditions for isolating the individual monoclinic (M) and triclinic (T) crystals.
